# Belonging to Socially Excluded Groups as a Predictor of Vaccine Hesitancy and Rejection

**DOI:** 10.3389/fpubh.2021.823795

**Published:** 2022-01-20

**Authors:** Yohanan Eshel, Shaul Kimhi, Hadas Marciano, Bruria Adini

**Affiliations:** ^1^Stress and Resilience Research Center, Tel Hai and University of Haifa, Haifa, Israel; ^2^Multinational Resilience and Well-Being Research Center, Tel Aviv University, Tel Aviv, Israel; ^3^Stress and Resilience Research Center, Tel-Hai College, The Institute of Information Processing and Decision Making (IIPDM), University of Haifa, Haifa, Israel; ^4^Department of Emergency and Disaster Management, Multinational Resilience and Well-Being Research Center, School of Public Health, Sackler Faculty of Medicine, Tel Aviv University, Tel Aviv, Israel

**Keywords:** vaccine hesitancy, vaccine rejection, conspiracy theories, partially excluded social groups, COVID-19

## Abstract

The scientific call for vaccination against the COVID-19 pandemic has met hesitancy, postponement, and direct opposition of parts of the public in several countries. Mistrusting the COVID-19 vaccine, distrusting the authorities, and unrealistic optimism, are three major reasons employed in justifying vaccine hesitancy. The present study examines two major issues. First, it strives to identify individuals that are unwilling to adhere to the vaccination process, more strongly question the effectiveness and necessity of the COVID-19 vaccine, and wonder about potential covert reasons for its administration. Second, it investigates associations between such “conspiracy” claims and the actual rejection of the vaccine. We assume that individuals belonging to social groups which are partly excluded by the general society will be less willing to fulfill the demands of this society, more inclined to reject the vaccine and associate it with some hidden conspiracy. A relatively large sample of the Israeli public (*N* = 2002) has responded to an anonymous questionnaire pertaining, among other things, to vaccine hesitancy and the individual level of vaccine uptake. Previous research has mainly examined the reasons for vaccine hesitancy. The present study's results indicate that three out of four social exclusion criteria (young adulthood, low level of income, and orthodox religiosity) have negatively predicted vaccine uptake and positively predicted three types of reasoning for vaccine hesitancy. Young adulthood was the strongest predictor of vaccine rejection. Attempts at convincing hesitating individuals to uptake this vaccine have often failed in many countries. As varied reasons underlie vaccine refusal, it is suggested that the approach to different vaccine rejecting groups should not be generic but rather tailor-made, in an attempt to influence their perceptions and behavior.

## Introduction

The COVID-19 pandemic has caught countries worldwide unprepared for coping with this plague and without a supply of an effective vaccine. Vaccines are considered one of the most successful public health interventions of the 20th century for containing infectious diseases (1). Recent data show that most of the inhabitants of Europe (2), North American (3, 4), and South American countries (5) are willing to be vaccinated against this pandemic. The majority of the Israeli population has already been inoculated, at least once, against the COVID-19 virus (6). However, despite the substantial risk of this pandemic, a substantial number of individuals all over the world express vaccine hesitancy and vaccine rejection. It should be noted that vaccine hesitancy is not a specific characteristic of the COVID-19 endemic. It is as old as the vaccine itself, and was also observed in previous pandemics [e.g., (7–10)].

Previous Israeli studies have found differences in vaccine hesitancy among health professionals (11, 12). The present study examines hesitancy in the general Israeli public and examines two major subjects. The major issue, which has hardly been examined empirically, refers to the impact of belonging to a socially excluded or partly excluded group, on vaccine rejection. In terms of Israel, “vaccine rejection” refers to one's status concerning the full vaccination process, which is required of Israeli citizens (i.e., to date, two vaccines and a booster). The second concern is vaccine hesitancy, which is expressed by questioning the necessity and effectiveness of this vaccine. These doubts frequently involve suspicions, leading to the perception that administering it to the public is associated with some kind of conspiracy.

A recent worldwide study explains COVID-19 vaccination hesitancy by mistrust in several key actors, including scientists, domestic healthcare professionals, and politicians (13). Additional research claims that this vaccine hesitancy often reflects conspiracy beliefs (14). These ideas have flourished with the COVID-19 pandemic, due to the spectacular rate of medical misinformation (15), and a growing readiness to accept statements from sources that question the legitimacy of the political system (16, 17). Conspiracy theories have been defined as “attempts to explain the ultimate causes of significant social and political events and circumstances, with claims of secret plots by two or more powerful actors” [(18), p. 4]. Heightened collective uncertainty and fear characteristics of social crises might enhance attempts to explain this threatening, complex and unpredictable situations, in terms of conspiracy beliefs (19, 20). Freeman and Bentall (21) claim that although false conspiracy theories are not supported by evidence, those who hold them believe that the present crisis is falsely presented by some unknown power, which presents the public with a cover-up narrative of the actual situation.

Attempts to understand the identity of those who regard vaccinations as involving a conspiracy of some unknown power, claim that less educated people hold these beliefs more often (22) and that individuals of lower-income and education, as well as those who regard themselves as politically powerless, are more susceptible to conspiracy theorizing about the origins and severity of the current pandemic (23). An additional review of 97 articles confirms that women, young adults, low education, and low-income individuals, as well as extremely religious and non-liberal people, are more prone to vaccine hesitancy (24). An Australian study (25) adds that living in disadvantaged areas and holding more populist views are associated as well with higher vaccine hesitancy.

In addition to the above already established findings concerning characteristics of vaccine hesitancy and vaccine dissenters, we suggest that in case of an epidemic, people who belong to social groups which are partly excluded and perhaps less appreciated, as well as those who deliberately choose to isolate themselves from this society, are more likely to believe in conspiracy claims. Furthermore, they are more likely to reject the vaccine aimed at coping with this plague. However, there is hardly any empirical data concerning the impact of being part of such a group on the decision of whether or not to be vaccinated.

In line with Douglas' (26) analysis of the functions of conspiracy and the identified characteristics of those who hold conspiracy ideas more readily (13), we assume that in many cases the conspiracy beliefs expressed in cases of vaccine hesitancy and rejection may have a distinct social function. These responses can constitute channels of the objection, employed by individuals who feel that they are either partly excluded from the general society, or are not well-assimilated within it.

The European Commission (27) has pointed out the objective risk factors, which may exert a negative influence on the prospect of social inclusion: low income, unskilled labor, poor health, low education level, school dropout, inequality, immigration, discrimination, and racism, old age, divorce, and living in a “problem accumulation area.” Rather than defining marginalization in such generality, we claim that belonging to a socially excluded, or partly excluded group constitutes a subjective lens through which people look at reality. In contrast, taking part in social interactions and feelings included helps people sustain their psychological well-being (28). Hence, those who feel socially excluded are likely to suffer aversive psychological consequences (29, 30). Theoretical analyses claim that relational evaluation is a key mechanism in understanding the degree to which such exclusion causes negative psychological outcomes, and promotes behaviors aimed at safeguarding this evaluation (31, 32).

We believe that in terms of the Jewish population of Israel, the individual sense of being segregated may be associated with belonging to the four following groups. Lower-income and lower education levels are two attributes that may make people feel that their chances of improving their living conditions are rather scarce and that they are already partly excluded by the general society (33). There is growing evidence that income inequality is associated with mental health outcomes and may cause status anxiety, clinical depression as well as a low self-perception (34). Ultra-orthodox religiosity, which promotes the disagreement on the issue of what Jewish identity is mainly about, constitutes a third potential exclusion reason. The orthodox perspective is that being Jewish is mainly a matter of religion, while the majority of secular Jews tend to regard Judaism mainly as a matter of ancestry and culture (35). Ultra-orthodox individuals wish, therefore, to exclude themselves from the secular way of life of the general society, and live as a separate social entity most likely in segregated and closed communities. Young adulthood may constitute a fourth reason for feeling exclusion. Young adults who are well aware of the fact that they have not as yet become a part of the grownup society, are likely to wonder how their lives will look like in the future, and whether they will succeed in establishing a desired social or professional position when they will grow (23). There is no clear definition for the developmental stage of young adulthood, but since its developmental tasks are attained at different stages, the consolidation of adult status is likely to be achieved closer to the end of the third decade of life (36). In line with this analysis, young adulthood is determined, in the present study, by the 20–39 years' age range.

The Israeli government currently demands all inhabitants to show good citizenship and social responsibility to fellow Israelis, by being vaccinated against the COVID-19 pandemic. We assume that individuals who belong to partly excluded social groups, as were presented in the above paragraphs, are more likely to express criticism of the integrity and the intentions of the authorities, as well as the pharmaceutical companies, and to feel that some conspiracy underlies the vaccination request. Furthermore, we expect them to respond negatively to this governmental request to complete their vaccination process.

The present study examines three modes of conspiracy claims in response to the vaccination request. First, suspect the authorities (37). Research has shown that conspiracy theories are likely to channel people's feelings of resentment toward political targets and to support radical attitudes (38). Second, questioning the integrity of the pharmaceutical companies: a general feeling of missing relevant information concerning the vaccine's effectiveness (39, 40), and concerns about unforeseen side effects and risks of this vaccine (2, 5). A third, indirect claim of conspiracy, which is phrased in terms of unrealistic optimism, argues that the risk of this plague, as presented by the authorities, is highly exaggerated and unjustified (41). Unrealistic optimism is a much wider concept which is defined as the “tendency for people to believe that they are less likely to experience negative events and more likely to experience positive events than are other people” [(42), p. 65]. In the present case, unrealistic optimists regard the threat of this pandemic as irrelevant to themselves, believing that they are more resilient than most people (43), and are less likely to experience negative events in general and to be infected by the COVID-19 virus, in particular.

Two hypotheses were examined:

- Younger age, lower education level, lower income, and a higher level of orthodox religiosity will negatively predict individual vaccine uptake and will positively predict the three modes of conspiracy claims (distrust in the authorities, distrust in the vaccine, and unrealistic optimism).
- Direct, as well as indirect, conspiracy claims concerning the COVID-19 vaccination will be positively correlated with each other, and will negatively correlate with individual vaccine uptake.

## Methods

### Data Collection

Individuals from all over Israel (*N* = 2002) have responded between October 8-12 2021 to an online questionnaire, distributed by an Internet Panel company that has a database of more than 65,000 panelists, representing the varied demographic groups in Israel (https://sekernet.co.il/). The respondents that are registered were approached directly by the company, without any disclosure of their identity to the researchers. To enable a representative sample, a stratified sampling method was employed, aligned with the data published by the Israeli Central Bureau of Statistics regarding geographic distribution, gender, and age. The study was approved by the Ethics Committee of the Tel Aviv University, #0003903-1 from September 30, 2021.

### Participants

Participants are 2002 individuals representing all parts of the Israeli Jewish population. Table 1 presents their demographic variables shows that their ages range from 18 to 82 years, 51% of them are females and 49% are males. They represent wide ranges of religiosity, income levels, political attitudes, and years of education. 68% of them have been vaccinated three times as requested.

**Table 1:** Demographic characteristics of the participants.

**Variable**		**Student sample**		
	**Group**	**Number**	**%**	**M (SD)**
Age	18–30	581	29	42.18 (15.64)
	40–31	441	22	
	50–41	366	18	
	51–60	298	15	
	61–82	316	16	
Gender	Men	985	49	
	Women	1,017	51	
Religiosity	Secular	927	46	1.84 (0.95)
	Traditional	640	32	
	Religious	266	13	
	Very religious	169	9	
Political attitudes	Extreme left	35	2	3.49 (0.89)
	Left	220	11	
	Center	706	35	
	Right	816	41	
	Extreme right	225	11	
Family income compared to average in Israel	Much below	532	27	
	Below	441	22	
	Average	597	30	
	Above	325	16	
	Much above	107	5	
Education	1. Elementary	31	2	3.33 (1.06)
	2. High school	488	24	
	3. Higher education	583	29	
	4. B.A.	580	29	
	5. M.A. and above	320	16	
Nationality	Jewish	1,880	94	
	Other	122	6	
Family status	Bachelor	541	27	
	Married	1,158	58	
	Divorce	169	8	
	Widower	27	1	
	In a relationship	107	5	
Vaccine status	1. Three vaccines	1,367	68	
	2. Two vaccines	315	16	
	3. One vaccine	98	5	
	4. No vaccine	222	11	

### Measures

#### Level of Vaccine Uptake

Israeli residents were requested, to date, to be vaccinated three times against COVID-19 (the third vaccine is a booster). The degree of vaccine uptake was determined by a single item: “To what extent are you currently vaccinated against the COVID-19?” The four-point response scale ranges from 1 = not vaccinated, to 4 = Vaccinated three times.

#### Concerns About Potential Conspiracies

This scale which has been devised for the present study includes three sub-scales. The first (eight items, Cronbach's α = 0.885) refers to a disbelief in the COVID-19 vaccine (examples: “There is not enough scientific support for the effectiveness of this vaccine”; “The COVID-19 vaccine prevents the human body from developing its natural antibodies”). The second sub-scale (three items, α = 0.730) pertains to disbelief in the authorities (examples: “The COVID-19 vaccine represents a conspiracy of the authorities”; “The COVID-19 vaccine is aimed at controlling and supervising people”). The third scale (four items, α = 0.872) pertains to unrealistic optimism (examples: “The doctors' warnings on the risk of the COVID-19 pandemic are exaggerated”; “I cope with health issues better than other people, therefore, I don't need this vaccine”). The 5-point response scale ranged from 1 = Do not agree at all, to 5 = Agree very much.

The five investigated demographic attributes were defined as follows:

*Young adulthood*. Respondents indicated their age in years.
*Religiosity* was determined by the question “How would you define your level of religiosity?” The four response options were: 1. Secular, 2. Traditional, 3. Religious, 4. Ultra-orthodox.
*Income level* was established by the following item: “The average income of an Israeli family today is 18,671 NIS per month. Your family's income is 1. Much lower than this average, 2. Lower than this average, 3. About this average, 4. Higher than this average, 5. Much higher than this average.”
*Political attitudes* were determined by the following item: “How would you define yourself politically as far as foreign affairs and security policies are concerned?” The five response options were: 1. Extreme left, 2. Left, 3. Center, 4. Right, 5. Extreme right.
*The level of education* was determined by the item “What is your education level?” The five response options were: 1. Primary education, 2. Secondary education, 3. Higher than secondary education (vocational), 4. Bachelor's degree, 5. Masters' degree or higher.

### Statistical Analysis

The hypotheses were examined by means of a path analysis/Amos Structural Equation Modeling, in which the four predictors and the four predicted variables were controlled for each other [IBM, SPSS, https://www.ibm.com/il-en/marketplace/structural-equation-modeling-sem; (44)]. Maximum likelihood estimates were employed and examined a saturated model, as we did not find any studies that supported an alternative model. It is important to note that in a saturated model, there is no need to examine a model fit as the default and the saturated model are the same (45). This saturated model (all paths are examined), which examined this hypothesis, included the four demographic attributes as the predictors; the three conspiracy expressions and the level of vaccination were the predicted variables. The variability in vaccine uptake according to demographic characteristics of vaccinated vs. none-vaccinated individuals was examined using *t*-test. All statistical analyses were performed using IBM SPSS and AMOS software version 26. *P*-values lower than 0.05 were considered as statistically significant.

## Results

Hypothesis A claimed that younger age, lower levels of education and income, as well as more orthodox religiosity will negatively predict vaccine uptake and will positively predict each of the three conspiracy expressions.

**Figure 1:**
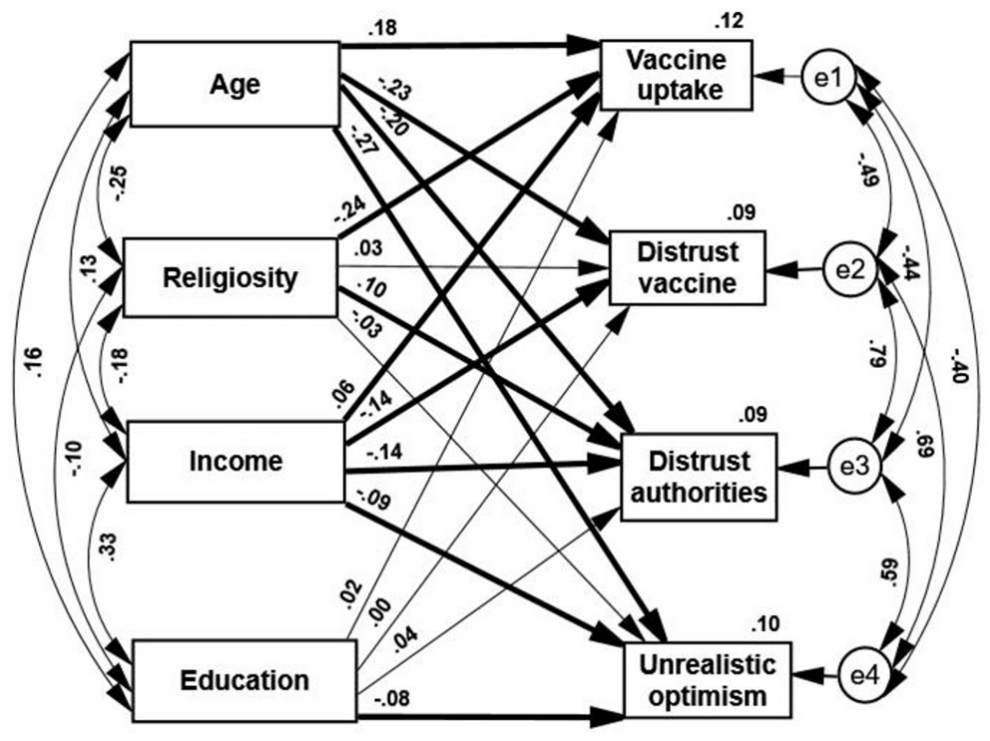
Standardized estimates of path analyses of demographic characteristics predicting vaccine hesitancy and uptake. Thin paths are insignificant. Thick path *p* < 0.01.

The path analysis indicated the following (Figure 1): (a) Age of the respondents was positively correlated with levels of education and income and negatively correlated with orthodox religiosity. This religiosity was negatively correlated with the level of income and with formal education. Income is positively correlated with the level of education.

(b) Three out of the four demographic attributes negatively predicted the vaccination status. Higher vaccine uptake was positively predicted by older age and higher income and negatively predicted by orthodox religiosity. It was not significantly predicted by the level of education. These results generally supported the first part of the first hypothesis.

(c) In line with the second part of this hypothesis, younger age and lower income positively and significantly predicted each of the three claims of covert intentions (distrusting the vaccine, mistrusting the authorities, and unrealistic optimism). Higher claims of mistrust were made by younger and low-income respondents. A lower level of education negatively and significantly predicted unrealistic optimism, and a higher level of religiosity positively predicted distrust in the authorities.

(d) Despite the differences among the three modes of vaccine rejection, they correlated positively with each other, indicating that all of them were likely connected to a more general source of conspiracy claims. In addition, all these three claims correlate negatively and significantly with vaccine uptake, thus can be viewed as attitudes that lead to action. Those who expressed a higher sense of conspiracy failed to complete their vaccination process to a greater extent.

A further examination of the variability of level of vaccination according to the demographic characteristics was done by computing *T*-tests which compared these attributes of the individuals who have been vaccinated three times, with those who did not vaccinate at all. Table 2, presenting these comparisons, shows that the vaccinated group surpasses the none-vaccinated group significantly on levels of education (medium effect size), age (large effect size) and income (medium effect size). The vaccinated group scores lower on level of religiosity (large effect size). No gender differences were found between the two groups. These findings constitute additional support for the path analysis results.

**Table 2:** *T*-tests comparing the demographic characteristics of individuals vaccinated three times vs. none-vaccinated individuals.

		**Not vaccinated** ***N*** **= 222**	**Vaccinated** ***N*** **= 1,367**	***t***	**Sig. (2-tailed)**	**Effect size** **Cohen's d**
Education	Mean	3.01	3.43	−5.810	0.000	0.40
	SD	0.989	1.068			
Gender	Mean	1.51	1.50	0.198	0.843	0.01
	SD	0.501	0.500			
Age	Mean	34.68	45.69	−12.050	0.000	0.78
	SD	12.027	15.823			
Religiosity	Mean	2.25	1.67	7.761	0.000	0.60
	SD	1.058	0.848			
Income	Mean	2.14	2.66	−6.090	0.000	0.46
	SD	1.164	1.197			

## Discussion

The present study examined the impact of belonging to a partly excluded social group on the level of vaccine uptake, and its association with perceived “conspiracy” theories. The study was conducted during October 2021, a period characterized by an ongoing decrease in levels of COVID-19 infectivity, and an increase in levels of vaccinations. The request for the COVID-19 vaccination raised strong public claims of some hidden conspiracies which were directed at the pharmaceutical companies and the political authorities. Previous research linked conspiracy beliefs with vaccination hesitancy (46) suggesting that conspiracy beliefs may undermine the motivation to take action in case of a pandemic (47). The World Health Organization (48) claimed that vaccine hesitancy was increased by the following causes: (1) people's belief that they are at low risk of contracting COVID-19, or that the consequences of becoming infected will not be severe; (2) people's lack of confidence in the vaccines' effectiveness and specific beliefs that the COVID-19 vaccine was rushed and not tested thoroughly; (3) the trust in the vaccine efficiency was undermined by the regulation to wear masks and to maintain social distancing despite being vaccinated; and, (4) skepticism about covert profit motives of pharmaceutical companies. Furthermore, people were inevitably exposed to misinformation, rumors, and a variety of false conspiracy theories, which could have eroded their confidence in the vaccine specifically and the vaccination program, in general.

The WHO (48) criteria for vaccine hesitancy referred to the general public. Several studies pointed at demographic characteristics which were associated with vaccine hesitancy and conspiracy ideas [e.g., (22, 23)]. These studies did not associate vaccine rejection with belonging to groups that were partly excluded from the general society, nor did they claim that these conspiracy ideas would be endorsed more readily by individuals who were part of such groups (49, 50). The present study clearly shows that belonging to any of the partly excluded young adults, low income, low education, or higher religiously orthodox groups, negatively impacted the vaccine uptake. Young adults, who may wonder whether they will succeed in establishing their desired social position in the future (23), are not generally considered as individuals whose place in society is still undetermined. They are not regarded as partly marginal like the low income group. However, it cannot be argued that young adults may be hesitant to get vaccinated against the COVID-19 due to relatively lower risks to their health, compared with older adults. This appears to be the case, despite the fact that the Israeli Ministry of Health (51) indicated contrarily, that the Israeli young adult age group (aged 20–39) has suffered a higher percentage of Coronavirus infections compared to the other age groups.

Previous research assumed that those who postponed being vaccinated would eventually reject this vaccine altogether [e.g., (14)]. The present data showed that vaccine hesitancy and questioning the effectiveness and necessity of the vaccine were indeed negatively correlated with vaccine uptake. We are not aware of a prior study that has demonstrated empirically a direct impact of being a part of such excluded social groups, on actual vaccine rejection. Our data showed further that belonging to one of these groups predicted higher rates of the three conspiracy claims as well, although less consistently.

The present study indicated that individual vaccine status, i.e., the actual level of vaccine uptake (out of the three required injections), was significantly predicted by belonging to a partly excluded social group and that being a young adult impacted most strongly vaccine rejection and hesitancy: the younger the age, the greater the hesitancy and rejection of this vaccine. What characterizes members of this group? Young adulthood requires the adoption of new roles and statuses and achievement of success in several domains concurrently: leaving the parental home to establish one's residence, gaining financial independence, completing school, progressing into full-time employment, getting married, and becoming a parent (52, 53). These actions emphasize the fragility of the process of personal development which is tested anew during young adulthood (54). The constant awareness of young adults of the assignments which lay ahead of them, and the vital importance of succeeding in them, constantly emphasize their sense of not being assimilated yet in the adult society (55–57). Furthermore, the present results indicate that two of the investigated demographic characteristics, constitute the best predictors of both vaccination uptake and hesitancy: young adulthood and the lower income.

## Limitations

The major limitation of this study is common to all studies which employ the self-report technique. We assume that the information provided by the participants is both sincere and exact since they are defended by anonymity. However, there is no way to test this assumption. Second, we have used short forms of the scales that were previously employed. Although these short scales have retained their high reliabilities, employing the full scales is still recommended. A third limitation is that the present study examines vaccination hesitancy and refusal only among Israeli Jews, who may feel excluded in part from the general society. Further research should investigate as well Israeli Arabs, who constitute a large Israeli minority, which is likely to feel partly excluded by the general public (58).

## Conclusion

One study of the public's attitudes toward the COVID-19 virus vaccine recommends creating an environment that will make the vaccine more available and increase social influence by using the recommendations of particularly trusted experts. The assumption is that these experts would increase the public's motivation and compliance by dialogues about the safety and benefits of this vaccine, as compared to the risks and uncertainty associated with it (48). Another analysis regards the belief in COVID-related conspiracy theories as to the source of the resistance to both preventive behaviors and future vaccines for this virus. It recommends a confrontation of both conspiracy theories and vaccination misinformation, to prevent further spread of the virus by politically conservative American outlets that have supported COVID-related conspiracy theories (23). The present study demonstrated empirically that belonging to a partly excluded social group negatively affected the COVID-19 vaccination. We believe, therefore, that in such cases of vaccine rejection more efficient approach would be to fit a tailor-made message to each specific group independently. Members of these groups could be encouraged to uptake the vaccine provided that they will be approached by trusted and respected group leaders with whom they may identify and whose messages they can accept. Each group should be approached differently. More sophisticated individuals would appreciate a presentation of the pros and cons concerning this vaccine, whereas other groups are more likely to prefer clear-cut information presented by an authority figure. Thus, for instance, there is reason to believe that more orthodox religious people will listen more readily to an orthodox religious authority figure, rather than to public health officials. Future studies should investigate the contribution of individual sense of social exclusion to vaccine rejection and the psychological means employed by members of socially excluded groups, that impact their adherence or rejection of the request to be vaccinated against COVID-19.

## Data Availability Statement

The raw data supporting the conclusions of this article will be made available by the authors, without undue reservation.

## Ethics Statement

The studies involving human participants were reviewed and approved by Ethics Committee of the Tel Aviv University. The patients/participants provided their written informed consent to participate in this study.

## Author Contributions

SK and BA conceived the study and collected the data. YE analyzed the data and wrote the first draft. HM and YE validated the data analysis and quality assurance. All authors reviewed and modified the paper.

## Conflict of Interest

The authors declare that the research was conducted in the absence of any commercial or financial relationships that could be construed as a potential conflict of interest.

## Publisher's Note

All claims expressed in this article are solely those of the authors and do not necessarily represent those of their affiliated organizations, or those of the publisher, the editors and the reviewers. Any product that may be evaluated in this article, or claim that may be made by its manufacturer, is not guaranteed or endorsed by the publisher.
